# Isoform-specific involvement of Brpf1 in expansion of adult hematopoietic stem and progenitor cells

**DOI:** 10.1093/jmcb/mjz092

**Published:** 2019-09-30

**Authors:** Qiuping He, Mengzhi Hong, Jincan He, Weixin Chen, Meng Zhao, Wei Zhao

**Affiliations:** 1 RNA Biomedical Institute, Sun Yat-sen Memorial Hospital, Sun Yat-sen University, Guangzhou 510120, China; 2 Key Laboratory of Stem Cells and Tissue Engineering, Sun Yat-sen University, Ministry of Education, Guangzhou 510080, China

**Keywords:** Brpf1 inhibitor, OF-1, Brpf1a, Mn1, hematopoietic stem and progenitor cell expansion

## Abstract

Bromodomain-containing proteins are known readers of histone acetylation that regulate chromatin structure and transcription. Although the functions of bromodomain-containing proteins in development, homeostasis, and disease states have been well studied, their role in self-renewal of hematopoietic stem and progenitor cells (HSPCs) remains poorly understood. Here, we performed a chemical screen using nine bromodomain inhibitors and found that the bromodomain and PHD finger-containing protein 1 (Brpf1) inhibitor OF-1 enhanced the expansion of Lin^−^Sca-1^+^c-Kit^+^ HSPCs *ex vivo* without skewing their lineage differentiation potential. Importantly, our results also revealed distinct functions of Brpf1 isoforms in HSPCs. Brpf1b promoted the expansion of HSPCs. By contrast, Brpf1a is the most abundant isoform in adult HSPCs but enhanced HSPC quiescence and decreased the HSPC expansion. Furthermore, inhibition of Brpf1a by OF-1 promoted histone acetylation and chromatin accessibility leading to increased expression of self-renewal-related genes (e.g. *Mn1*). The phenotypes produced by OF-1 treatment can be rescued by suppression of Mn1 in HSPCs. Our findings demonstrate that this novel bromodomain inhibitor OF-1 can promote the clinical application of HSPCs in transplantation.

## Introduction

Hematopoietic stem cells (HSCs) are the most extensively studied stem cells with proven clinical utility (Li and Clevers, 2010). HSC transplantation has the potential to treat various diseases, including immunodeficiency, hematological malignancies, and other types of cancer (Copelan, 2006; Mantel et al., 2015). The lack of HLA-matched donors presents a serious limitation to allogeneic HSC transplantation. While umbilical cord blood (UCB) may one day become an alternative source of HSCs, the number of HSCs in UCB is often too low for successful transplantation (Czechowicz et al., 2007). Therefore, *ex vivo* hematopoietic stem and progenitor cell (HSPC) expansion would greatly improve clinical availability of transplantation therapies (Fares et al., 2014).

The regulation of HSC self-renewal remains a fundamental question related to *ex vivo* HSPC expansion. Previous studies have identified multiple key intrinsic factors in regulation of HSC self-renewal, including chromatin-associated factors (e.g. Bmi-1 and MOZ) (Hosen et al., 2007; Sheikh et al., 2016) and transcription factors (TFs, e.g. Runx1 and Meis1) (Kumano and Kurokawa, 2010; Cai et al., 2012). Moreover, numerous investigations have shown that signals from the HSC niche are crucial to the regulation of HSC self-renewal and differentiation (Liu et al., 2019). The number of HSCs in the niche is determined by the frequency of HSC self-renewal, which leads to the generation of two stem/progenitor cells, relative to the frequency of differentiation. The relative frequency of these events creates a balance between HSC self-renewal and differentiated daughter cell generation. There is an active HSC differential proliferation during fetal blood development (Sigurdsson et al., 2016). In adulthood, HSCs are generally quiescent in the niche, whereas diverse stimuli can trigger self-renewal and cause cells to enter into the cell cycle (Bernitz et al., 2016). However, the induced proliferation is often associated with DNA damage and apoptosis (Dawar et al., 2016). *Ex vivo* expansion thus requires approaches that result in increased self-renewal without further differentiation and apoptosis. Importantly, the mechanisms by which mammalian HSCs undergo self-renewal in fetal liver during development and in adulthood are different. Improved understanding of the regulation of genes associated with quiescence, self-renewal, proliferation, and differentiation in adult HSCs would help achieve HSPC *ex vivo* expansion.

Lysine acetylation of histone proteins is a critical modification that regulates chromatin structure, promotes gene transcription, and may play a role in HSC self-renewal and differentiation (You et al., 2016; Hua et al., 2017; Valerio et al., 2017). Bromodomain proteins, which can be categorized by their structural domains and divided into bromodomain and extra-terminal (BET) or non-BET families, specifically bind to histone acetylation marks. The BET subfamily, which includes BRD2, BRD3, BRD4, and BRDT, specifically recognizes acetylation markers along H3 and H4 histone tails, activating transcription (Lambert et al., 2019). Inhibitors of BET proteins suppress proliferation and gene expression in embryonic stem cells (ESCs) (Di Micco et al., 2014), but BRD4 is dispensable for self-renewal and pluripotency of ESCs (Rodriguez et al., 2014; Finley et al., 2018). Early clinical trials of BET inhibitors have shown promise, especially in acute myeloid leukemia (Lucas and Gunther, 2014; Gerlach et al., 2018). Similar to BET family proteins, the non-BET proteins have been associated with various cancers as well as with developmental disorders (Hugle et al., 2017). Recent publications have demonstrated that non-BET bromodomains can also be specifically targeted by chemicals (Theodoulou et al., 2016). However, the phenotypic consequences of HSC self-renewal and differentiation mediated by BET or non-BET inhibitors have yet to be reported.

Here, we show that histone acetylation on master TFs contributes to HSC self-renewal and differentiation. We demonstrate that the Brpf1 inhibitor OF-1 increases the number and proportion of functional HSPCs (Lin^−^Sca-1^+^c-Kit^+^ cells, LSKs) by modulating histone acetylation and chromatin accessibility of HSC self-renewal-related genes, such as *Mn1*. Moreover, our results revealed distinct functions of Brpf1 isoforms in LSKs. These observations support a role for histone acetylation and bromodomain proteins in HSC self-renewal and identify a new approach for enhancing *ex vivo* expansion of HSPCs.

## Results

### Non-BET bromodomain inhibitor OF-1 enhances expansion of LSKs

**Figure 1:**
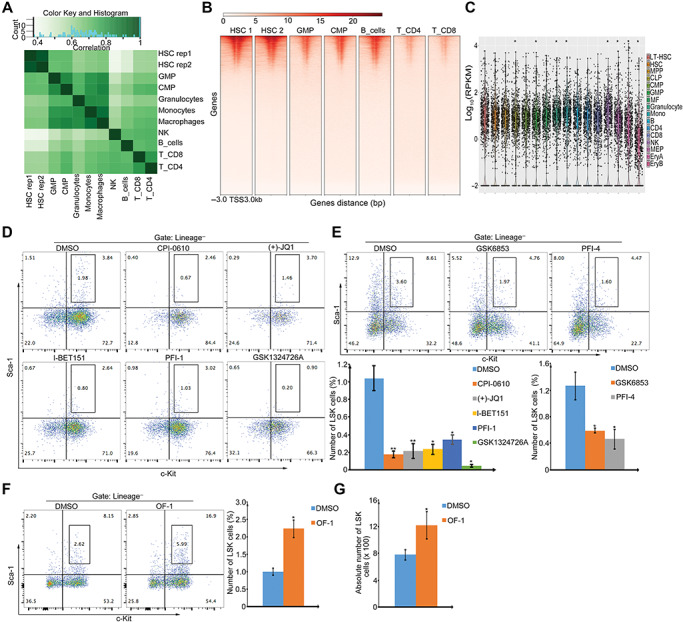
Non-BET bromodomain inhibitor OF-1 promotes expansion of cultured LSKs. (**A**) Hierarchical clustering of H3K27ac (data from GSE60103) analyzed in HSCs (HSC: Lin^−^, c-Kit^+^, Sca-1^+^, Flk2^−^, CD34^−^), granulocyte–macrophage progenitor cells (GMP: Lin^−^, c-Kit^+^, Sca-1^+^, FcgRII^high^, CD34^+^), common myeloid progenitor cells (CMP: Lin^−^, c-Kit^+^, Sca-1^+^, FcgRII^low^, CD34^+^), granulocytes, monocytes, macrophages, B cells, natural killer cells (NK), CD4^+^ T cells (T_CD4), and CD8^+^ T cells (T_CD8). Green reflects correlation index. (**B**) The heatmap showing the H3K27ac genome-wide distribution and signal intensity of H3K27ac peaks in HSC, GMP, CMP, B cells, T_CD4, and T_CD8. Each horizontal line represents the normalized signal intensity for a gene over its transcription start site (TSS). A ± 3 kb window is shown for each TSS. The colored scale bar shows the relative binding intensity. (**C**) Box plot depicting the expression of top 1000 genes in long-term HSC (LT-HSC), HSC, multipotent progenitor (MPP), common lymphoid progenitor (CLP), CMP, macrophage (MF), granulocyte, monocyte, B cell, CD4^+^ T cell, CD8^+^ T cell, NK, megakaryocyte–erythrocyte progenitor (MEP), erythrocyte A (EryA), and erythrocyte B (EryB). Data are from GEO dataset (GSE60101) with statistical analysis defined by Wilcoxon signed-rank test. *P < 0.05; **P < 0.01. (**D–F**) Percentage of LSKs in gated cells (Lin^−^) upon treatment with 1 μM BET inhibitors (CPI-0610, (+)-JQ1, I-BET151, PFI-1, and GSK1324726A; **D**), 1 μM non-BET inhibitors (GSK6853 and PFI-4 ; **E**), or 10 μM OF-1 (**F**). (**G**) Absolute number of LSKs in cultured cells upon 10 μM OF-1 treatment. Data represent mean ± SD from three independent experiments, with statistical analysis defined by two-tailed Student’s *t*-test. *P < 0.05; **P < 0.01.

To investigate dynamic changes in the histone acetylation that control gene expression during HSC self-renewal and differentiation, we analyzed published chromatin immunoprecipitation sequencing (ChIP-seq) datasets (GSE60103) (Lara-Astiaso et al., 2014) for histone 3 lysine 27 acetylation (H3K27ac) in HSCs and in differentiated hematopoietic cells. Unsupervised hierarchical clustering analysis, which was based on the acquisition and loss of H3K27ac loci, clearly distinguished HSC from differentiated hematopoietic cells (Figure 1A). Comparison of the H3K27ac among HSC and differentiated cells revealed that H3K27ac loci were downregulated with differentiation (Figure 1B). We further revealed that genes associated with high H3K27ac in HSC were, as a group, highly expressed in HSCs and progenitor cells (Figure 1C).

Based on the fact that H3K27ac modifications create docking sites for bromodomains, the effects of small-molecule bromodomain inhibitors, including BET and non-BET bromodomain inhibitors (Supplementary Table S1), were screened by determining the proportion of LSKs from primary mouse bone marrow after expansion with the inhibitor. Three rounds of screening revealed that OF-1, a compound identified as a Brpf1 (non-BET) inhibitor, significantly increased LSK proportion (Figure 1D–F), and thus may improve HSPC expansion *ex vivo*. The LSK frequency of OF-1-treated cultures at Day 7 was twice that of the DMSO control culture. Other Brpf1 inhibitors, GSK6853 and PFI-4, suppressed LSK frequency compared to the dimethyl sulphoxide (DMSO) control, suggesting that OF-1 promotes HSC expansion through a different mechanism than GSK6853 and PFI-4. Determining the absolute numbers of LSKs confirmed the effect of OF-1 on proliferation when compared with control (Figure 1G).

### OF-1 attenuates differentiation and promotes expansion of cultured LSKs

**Figure 2:**
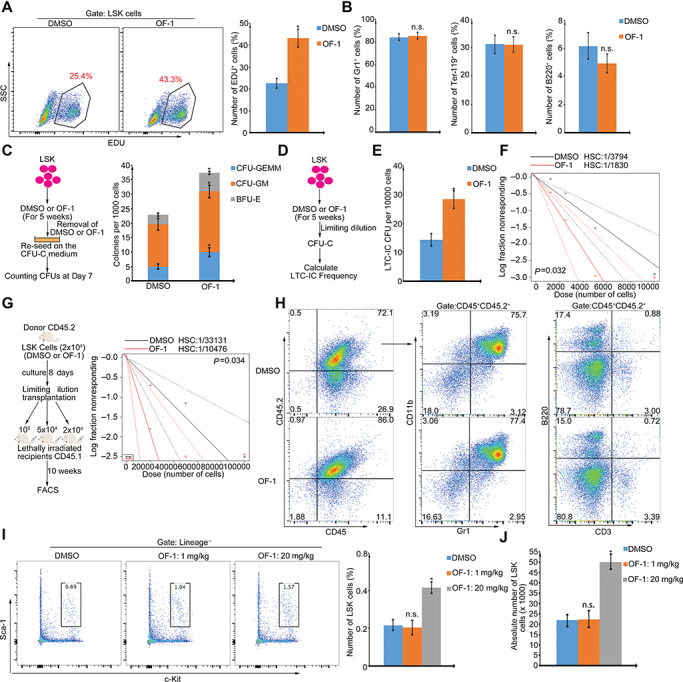
OF-1 facilitates the production of more functional LSKs *ex vivo* and *in vivo*. (**A**) EdU incorporation assay for proliferation for LSKs with or without 10 μM OF-1 treatment. (**B**) 10 μM OF-1 or DMSO were removed from HSC differentiation culture medium at Day 5. Composition of myeloid (Gr1^+^), B (B220^+^), and erythroid (Ter-119^+^) cells were analyzed at Day 7. (**C**) The *ex vivo* expanded cells with or without 10 μM OF-1 were reseeded on the methylcellulose-based medium for CFU assays. Erythroid progenitors (BFU-E), granulocyte–macrophage progenitors (CFU-GM), multi-potential granulocytes, erythroids, macrophages, and megakaryocyte progenitors (CFU-GEMM) were analyzed for colony-forming ability at Day 7. (**D**) The experimental scheme for LTC-IC assay. (**E**) LTC-IC assay using DMSO or OF-1-treated LSKs. The results are expressed as total number of CFU-C normalized to 10000 cells plated. (**F**) LDA to determine the LTC-IC frequency by extreme limiting dilution analysis (ELDA) at 5 weeks after 10 μM OF-1 or DMSO treatment. Dashed lines indicate 95% confidence interval. (**G**) Experimental scheme for ELDA to determine the frequency of functional HSCs at 8 days after 10 μM OF-1 or DMSO treatment. Dashed lines indicate 95% confidence interval (*n* = 6). (**H**) Percentage of donor (CD45.2)-derived CD45^+^ cells and different lineages (e.g. CD3^+^ T cells, B220^+^ B cells, and Gr1^+^CD11b^+^ myeloid cells). (**I** and **J**) Percentage (**I**) and absolute number (**J**) of LSKs in mice bone marrow with or without OF-1 i.p. injection at Day 10. Data represent mean ± SD from three independent experiments, with statistical analysis defined by two-tailed Student’s *t*-test. *P < 0.05; n.s., not significant.

Increased hematopoietic cell proliferation may improve long-term engraftment. To determine the effects of OF-1 on LSK cell proliferation, the fraction of actively dividing cells was measured by incorporating 5-ethynyl-2′-deoxyuridine (EdU) in cells *in vitro*. Over three days, a higher frequency of proliferating cells in OF-1-treated LSKs was observed (EdU^+^; ~ 43% in OF-1 vs. ~ 25% in DMSO) (Figure 2A). Control cultures contained a high frequency of Lin^+^ (differentiated cells), whereas OF-1-treated cultures contained mostly undifferentiated cells (Supplementary Figure S1A). OF-1 consistently inhibited colony-forming units of granulocytes, erythrocytes, macrophages, and megakaryocytes when culturing LSKs in differentiation medium (Supplementary Figure S1B). Therefore, OF-1 treatment favors self-renewing division, not differentiating division. Importantly, after removal of OF-1, the undifferentiated OF-1-treated cells generated comparable frequency of differentiated cells (Figure 2B) and more granulocyte, erythrocyte, macrophage, and megakaryocyte colonies compared with DMSO control (Figure 2C). To further demonstrate this point, we performed a quantitative limiting dilution assay (LDA) to evaluate the frequency of long-term culture-initiating cells (LTC-ICs), a retrospective assessment of the presence of functional HSPCs via *ex vivo* propagation. After long-term culturing, we found that OF-1 increased LTC-IC readout (Figure 2D–F). We conducted a LDA by transplanting 1 × 10^5^, 5 × 10^4^, or 2 × 10^4^ donor LSKs (from CD45.2 mice) into lethally irradiated CD45.1 recipient mice. Consistent with the increased cell number of HSPCs upon OF-1 treatment, we found that HSC frequency was increased ~3-fold compared with DMSO controls (Figure 2G). As shown in Figure 2H, the donor LSKs successfully reconstituted the different hematopoietic lineages (e.g. T cells, B cells, and myeloid cells) in recipient mice. Finally, we found that OF-1 treatment also promoted the *in vivo* proliferative potential of LSKs, indicated by both the increased frequency and absolute number of LSKs (Figure 2I and J).

### Brpf1a is the cellular target of OF-1 in cultured LSKs

**Figure 3:**
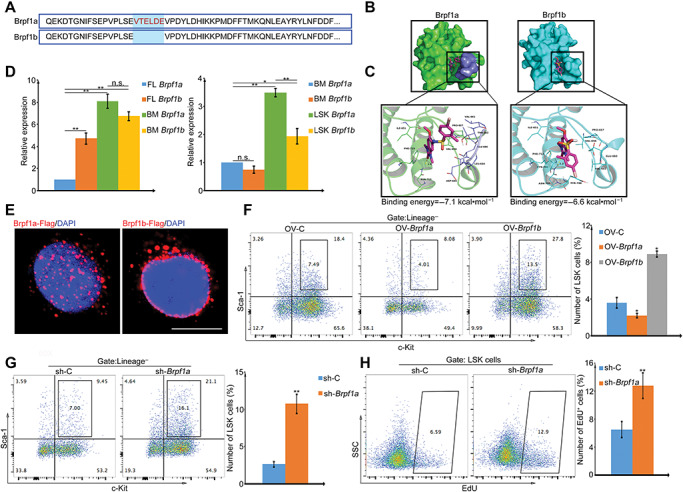
Brpf1a is the cellular target of OF-1. (**A**) Sequence alignment of Brpf bromodomains. In Brpf1a, six residues EVTELD (red) are inserted into the ZA loop. (**B**) OF-1 is docked into the binding site of Brpf1a (left, amino acids 661–666 of Brpf1a are colored in slate) or Brpf1b (right) protein. (**C**) Detailed view of the binding sites of OF-1–Brpf1a (left) and OF-1–Brpf1b (right). (**D**) Quantitative real-time polymerase chain reaction (qPCR) analysis of *Brpf1a* or *Brpf1b* mRNA relative expression in E14.5 fetal liver (FL) cells, 6–8 week bone marrow (BM) cells, and LSKs. (**E**) Immunofluorescence of Brpf1a or Brpf1b with the antibody against Flag (red) and DAPI (blue) in 293T cells transfected with Brpf1a-Flag or Brpf1b-Flag. Scale bar, 10 μm. (**F**) Percentage of LSKs with Brpf1a or Brpf1b overexpression after 7 days of culture. (**G**) Percentage of LSKs with *Brpf1a* knockdown (KD) after 7 days of culture. (**H**) EdU incorporation analysis of *Brpf1a* KD LSK cell proliferation. Data represent mean ± SD from three independent experiments, with statistical analysis defined by two-tailed Student’s *t*-test. *P < 0.05; **P < 0.01; n.s., not significant.

OF-1 is a pan-Brpf bromodomain inhibitor with relative strong selectivity for Brpf1 (Igoe et al., 2017; Meier et al., 2017). Previous reports showed that the longer Brpf1 isoform, Brpf1a, harbors a six-residue insert in the ZA-loop that prevented binding to histone peptides (Hibiya et al., 2009). To reveal the different preferences of OF-1 to Brpf1 isoforms, a molecular docking-based calculation was used to identify putative OF-1-binding sites in Brpf1a and Brpf1b proteins (Figure 3A). Based on binding energy calculation, a preference for OF-1 binding to the bromodomain of Brpf1a over Brpf1b was predicted. The 1,3-dimethyl-1,3-dihydro-2H-benzo[d]imidazol-2-one group of OF-1 stretched into the hydrophobic pocket of Brpf1a, comprising Ile-651, Val-656, and Phe-719, while the 4-bromo-2-methylbenzenesulfonamide group of OF-1 located at separate hydrophobic pocket of Brpf1a, surrounded by the residues Pro-657, Val-661, and Leu-664, forming stable hydrophobic association (Figure 3B and C).

Regulation of gene transcription is known to differ between fetal HSCs and adult HSCs. Notably, previous studies have implied that fetal HSCs have a distinct dependency on Brpf1 (You et al., 2016). Given our results showing disparate Brpf1 isoform inhibition by OF-1 in adult LSKs, we examined Brpf1a and Brpf1b expression in fetal and adult LSKs (Figure 3D). Brpf1b expression was significantly higher than Brpf1a in fetal liver. However, Brpf1a became the most abundant isoform in adult LSK cell populations, indicating that a competitive binding of Brpf1 isoforms with histone may present in adult HSCs (Figure 3D). The increased Brpf1a expression led us to examine whether Brpf1a functions differently from Brpf1b in adult LSKs. We found that Brpf1a was located at euchromatin region of nuclei, whereas Brpf1b was enriched at heterochromatin region against the nuclear envelop (Figure 3E). We next compared the effects of overexpression of Brpf1a and Brpf1b (Supplementary Figure S2A) on LSK cell expansion. The results showed that overexpression of Brpf1b increased the frequency of LSKs by 2-fold (Figure 3F). By contrast, overexpression of Brpf1a suppressed LSK frequency and number (Figure 3F). Targeted shRNA knockdown assays were performed to validate Brpf1a function in LSKs (Supplementary Figure S2B). Consistently, the LSK proportion increased significantly in sh-*Brpf1a* cells compared with sh-Control cells (Figure 3G). Furthermore, cellular proliferation assays showed a significant increase of EdU uptake in sh-*Brpf1a* LSKs compared with control LSKs (Figure 3H). All these data confirm that Brpf1a and Brpf1b function differently in regulating HSPC expansion *ex vivo*.

### Brpf1a depletion facilitates self-renewal-related gene expression in adult LSKs

**Figure 4:**
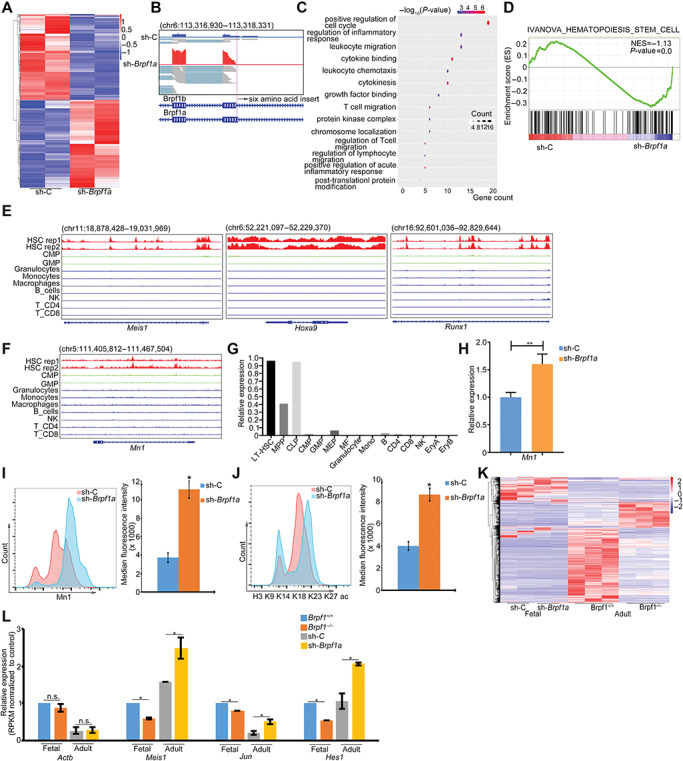
*Brpf1a* KD promotes self-renewal-related gene expression in adult HSCs. (**A**) The heatmap showing differentially expressed genes of LSKs with *Brpf1a* KD compared to control. (**B**) Integrative Genomics Viewer (IGV) showing the RNA-seq peaks of *Brpf1* mRNA. (**C**) GO analysis of differentially expressed genes of LSKs with *Brpf1a* KD compared to control. Color of circles denotes –log10 *P*-value and dot size represents gene counts. (**D**) GSEA reveals enrichment of the HSC signature in *Brpf1a* KD-regulated genes of LSKs. (**E** and **F**) IGV showing the H3K27ac ChIP-seq peaks at indicated gene loci. Data are from GEO dataset (GSE60101). X-axis shows genome position and Y-axis shows ChIP-seq signal. (**G**) *Mn1* mRNA expression in indicated cells from GEO dataset (GSE60101). (**H**) qPCR analysis of *Mn1* mRNA expression in *Brpf1a* KD and control LSKs. (**I**) Flow cytometry analysis of Mn1 protein level in *Brpf1a* KD and control LSKs. (**J**) Flow cytometry analysis of pan-histone acetylation (H3K9/K14/K18/K23/K27 ac) levels in *Brpf1a* KD and control LSKs. (**K** and **L**) The heatmap (**K**) and represented (**L**) differentially expressed genes of adult LSKs and E14.5 fetal liver cells. Data represent mean ± SD from three independent experiments, with statistical analysis defined by two-tailed Student’s *t*-test. *P < 0.05; **P < 0.01; n.s., not significant.

To define the role of Brpf1a in LSK gene regulation, we performed RNA sequencing (RNA-seq) to profile transcriptomes of LSKs infected with *Brpf1a* shRNA or control shRNA (Figure 4A). As expected, *Brpf1a* shRNA specifically reduced RNA-seq reads mapping to Brpf1a (Figure 4B). Intriguingly, Brpf1b expression increased in LSKs upon *Brpf1a* KD (Figure 4B). Gene ontology (GO) analysis of Brpf1a target transcripts revealed enrichment of genes related to cell cycle or inflammation, suggesting the involvement of Brpf1a in the regulation of hematopoiesis (Figure 4C). When we related our RNA-Seq data to the previously reported HSC gene sets by gene set enrichment analysis (GSEA), we found that, relative to control, *Brpf1a* KD LSKs were enriched with HSC signature genes (Figure 4D; Supplementary Figure S3A).

By analyzing H3K27ac profile during HSC differentiation, we found that H3K27ac at HSC signature genes *Meis1, Runx1, and Hoxa9* were markedly downregulated during HSC differentiation (Figure 4E). In addition, we found increased H3K27ac at lineage-specific hallmark genes *Cd3*, *S100a8*, and *Pou2af1* during HSC differentiation (Supplementary Figure S3B). H3K27ac at *Meningioma 1 (Mn1)*, whose overexpression has been found to modulate HSC proliferation (Carella et al., 2007), was highly enriched and positively associated with increased mRNA expression of *Mn1* in HSCs (Figure 4F and G). Moreover, *Mn1* mRNA expression increased markedly upon *Brpf1a* KD (Figure 4H). Consistently, fluorescence activated cell sorter (FACS) analysis validated that *Brpf1a* KD promoted the expression and histone acetylation of Mn1 in LSKs (Figure 4I and J). In addition, repression of Brpf1a expression in LSKs leads to increased expression of multiple genes critical for HSC expansion (Supplementary Figure S3C).

To further elucidate the role of Brpf1a in adult HSC gene transcription, we compared our *Brpf1a* KD RNA-seq in adult LSKs with *Brpf1* knockout (KO) RNA-seq in fetal HSCs (Figure 4K). Genes previously reported to be downregulated in *Brpf1* KO fetal HSCs, including *Meis1*, *Jun*, and *Hes1*, were upregulated in *Brpf1a* KD adult HSCs (Figure 4L). Thus, these data indicate that Brpf1a inhibition resulted in activation of a panel of self-renewal-related genes in adult HSCs, which were quite different from Brpf1 target genes in fetal liver HSCs.

### Inhibition of Brpf1a by OF-1 promotes expression of self-renewal-related genes in LSKs

**Figure 5:**
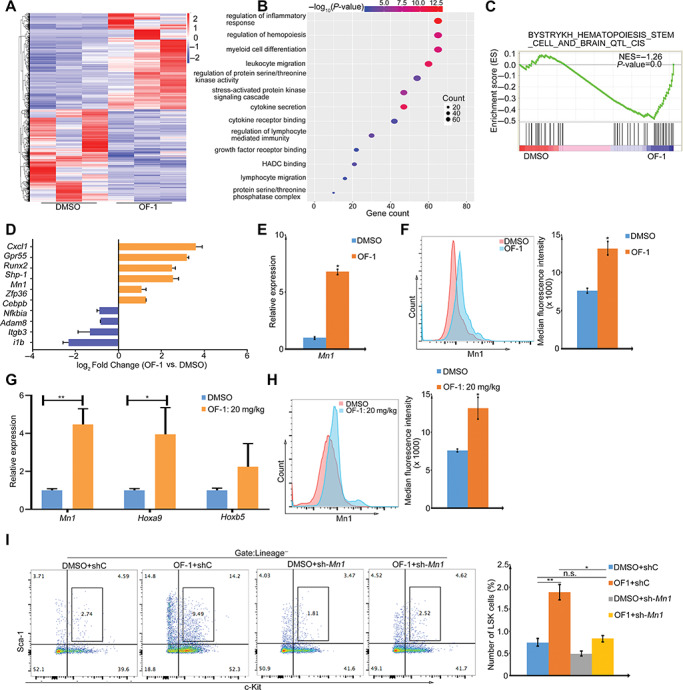
OF-1 promotes the expression of self-renewal-related genes in HSCs. (**A**) The heatmap showing differentially expressed genes of LSKs with or without 10 μM OF-1 treatment at Day 7. (**B**) GO analysis of differentially expressed genes of RNA-seq heatmap. Color of circles denotes –log10 *P*-value and dot size represents gene counts. (**C**) GSEA reveals enrichment of the HSC signature in OF-1-induced regulated genes of LSKs. (**D**) Relative mRNA expression levels of represented genes related to HSC self-renewal and differentiation of LSKs with 10 μM OF-1 treatment. (**E**) qPCR analysis of *Mn1* mRNA level of LSKs with 10 μM OF-1 treatment compared to DMSO treatment. (**F**) Flow cytometry analysis of Mn1 protein level from LSKs with DMSO or OF-1 treatment. (**G**) qPCR analysis of *Mn1, Hoxa9,* and *Hoxb5* mRNA levels of LSKs from 20 mg/kg OF-1-treated (i.p.) or DMSO control mice. (**H**) Flow cytometry analysis of Mn1 protein level of LSKs from 20 mg/kg OF-1-treated (i.p.) or DMSO control mice. (**I**) Percentage of LSKs after *Mn1* KD with or without 10 μM OF-1 treatment at Day 7. Data represent mean ± SD from three independent experiments, with statistical analysis defined by two-tailed Student’s *t*-test. *P < 0.05; **P < 0.01; n.s., not significant.

To define the underlying molecular mechanisms of Brpf1a inhibitor OF-1-mediated LSK cell expansion, we performed RNA-seq on isolated LSKs treated with or without OF-1. OF-1 treatment results in self-renewal-related genes such as *Runx1* and *Mn1* increased gene expression when compared with the DMSO control (Figure 5A). GO analysis revealed that inflammatory response was among the most deregulated pathways upon OF-1 treatment (Figure 5B). Importantly, relative to untreated LSKs, GSEA revealed a marked upregulation of HSC signature genes (Figure 5C), including *Mn1* and *Shp-1* (Figure 5D). qPCR and FACS analysis were used to validate upregulation of Mn1 (Figure 5E and F).

Previous studies have shown that Brpf1 interacts with MOZ through the MYST domain and enhances MOZ acetyltransferase activity, thereby promoting further histone acetylation (Perez-Campo et al., 2013). Besides its activity as a histone acetyltransferase (HAT), MOZ cooperates with Pu.1 and Runx1/2, playing a crucial role in hematopoiesis that is independent of Brpf1 (Supplementary Figure S4A). Also, the self-renewal-related TFs (e.g. *Runx1/2, Hoxa9, gata2*, and *Mn1*) showed decreased expression in *Moz* KO cells (Supplementary Figure S4B), suggesting that MOZ plays a critical role in the upregulation of hematopoiesis and self-renewal-related genes found in the OF-1-treated LSKs.

Next, we sought to determine whether OF-1 increased up-regulation of self-renewal-related genes *in vivo* (Figure 5G). LSKs derived from OF-1-treated mice consistently showed up-regulated Mn1 compared with DMSO controls (Figure 5H). We induced *Mn1* silencing (Supplementary Figure S4C) and observed that the percentage of LSKs remained stable after OF-1 treatment (Figure 5I), suggesting that Mn1 is necessary for HSPC expansion *ex vivo*.

### OF-1 potentiates chromatin accessibility at LSK self-renewal-related genes via modulation of histone acetylation

**Figure 6:**
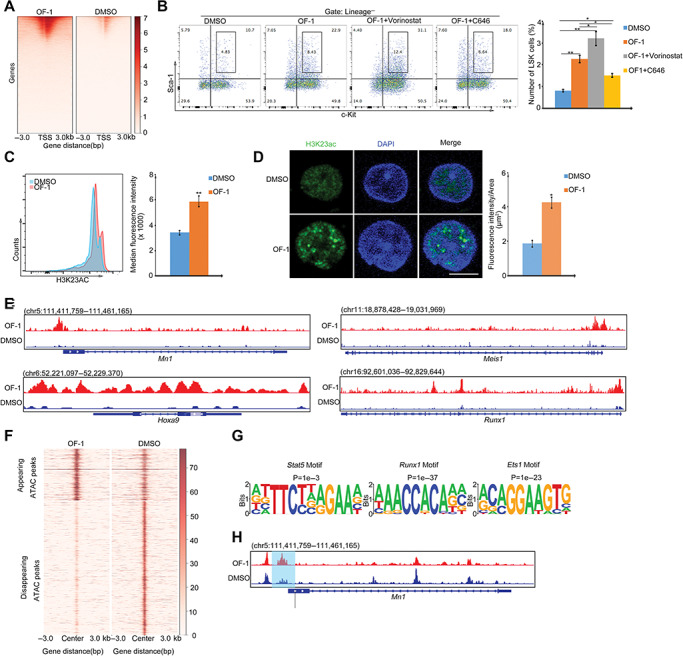
OF-1 regulates chromatin accessibility at self-renewal-related genes via modulation of histone acetylation in LSKs. (**A**) The heatmap showing the genome-wide distribution and signal intensity of pan-histone acetylation (H3K9/K14/K18/K23/K27 ac) in DMSO or 10 μM OF-1-treated LSKs. Each horizontal line represents the normalized signal intensity for a gene over its TSS. A ± 3-kb window is shown for each TSS. The colored scale bar shows the relative binding intensity. (**B**) Percentage of LSKs treated with 10 μM OF-1 or/and 0.1 μM Vorinostat/C646. (**C**) Flow cytometry analysis of H3K23ac levels of LSKs treated with DMSO or 10 μM OF-1. (**D**) Immunofluorescence staining of H3K23ac (green) of LSKs treated with DMSO or 10 μM OF-1. Nuclei are stained with DAPI (blue). Quantification of fluorescence intensity of H3K23ac of LSKs is shown on the right. Scale bar, 10 μm. (**E**) ChIP-seq peaks of pan-histone acetylation (H3K9/K14/K18/K23/K27 ac) at indicated self-renewal gene loci of LSKs treated with DMSO or 10 μM OF-1. X-axis shows genome position and Y-axis shows ChIP-seq signal. (**F**) ATAC-seq profiles showing appearing and disappearing peaks of LSKs treated with DMSO or 10 μM OF-1. Each horizontal line represents the normalized signal intensity for a peak over its center (upstream 3 kb and downstream 3 kb). The colored scale bar shows the relative binding intensity. (**G**) DNA motifs enriched in ATAC-seq peaks by HOMER Known motif analysis. (**H**) IGV showing ATAC-seq peaks at *Mn1* locus. The shadow indicates the increased chromatin accessibility upon OF-1 treatment. X-axis shows genome position and Y-axis shows ChIP-seq signal. Data represent mean ± SD from three independent experiments, with statistical analysis defined by two-tailed Student’s *t* -test. *P < 0.05; **P < 0.01.

We hypothesize that LSKs dominate Brpf1 isoform. Brpf1a blocks Brpf1b-mediated histone acetylation. To test whether Brpf1a inhibition by OF-1 modulates the self-renewal-related genes identified by RNA-seq through histone acetylation, we assessed pan-histone acetylation modification (H3K9/14/18/23/27ac) by ChIP. ChIP-seq analysis revealed that histone acetylation marks at proximal promoters markedly increased in LSKs upon OF-1 treatment (Figure 6A). To determine whether the activation of histone acetylation in OF-1-treated LSKs contributes to self-renewal and subsequent functional enhancement, OF-1-treated LSKs were exposed to the small-molecule HDAC inhibitor Vorinostat or HAT inhibitor C646. C646 partially abolished the expansion of LSKs induced by OF-1 treatment; however, Vorinostat augmented the OF-1 effects on LSK expansion (Figure 6B). Though it has been demonstrated that Brpf1 is required for H3K23ac in fetal HSCs, specific analysis of H3K23ac using FACS and immunostaining illustrated the stronger occupancy of H3K23ac in OF-1-treated adult LSKs compared with DMSO controls (Figure 6C and D). In addition, *Mn1, Runx1, Meis1*, and *Hoxa9* showed increased occupancy of histone acetylation (Figure 6E).

Furthermore, an assay for transposase-accessible chromatin by sequencing (ATAC-seq) in LSKs following OF-1 treatment showed dramatic change in global ATAC-seq signals (Figure 6F). Motif scanning using i-cisTarget revealed that these upregulated peaks were highly enriched for *Runx1, Stat5*, and *ETS1* motifs (Figure 6G). We observed significantly increased DNA accessibility at the promoters of self-renewal-related genes, such as *Mn1* (Figure 6H). We also observed unchanged ATAC-seq signals at the promoters of these lineage differentiation genes (Supplementary Figure S5), suggesting that OF-1 treatment did not affect differentiation potential of these LSKs. Together, ATAC-seq data support a crucial role for OF-1 in modulating chromatin structure to promote expression of self-renewal-related genes in HSCs.

## Discussion

Acetylated histones are important epigenetic signatures that dictate gene expression in various stages of normal hematopoiesis. In this study, we showed that intervention with the histone acetylation reader Brpf1, a bromodomain protein, affects regulation histone acetylation of a set of self-renewal-related genes. This reversible epigenetic effect could lead to chromatin accessibility enhancement and an active transcriptional pattern, thereby promotes *ex vivo* HSPC expansion without affecting HSPC differentiation potential. Moreover, we showed that Brpf1 isoforms have distinct functions in HSCs. Brpf1b but not Brpf1a is expressed at meaningful levels during fetal hematopoiesis and promotes HSC expansion, whereas Brpf1a becomes the dominant isoform in adult HSCs function in HSC quiescent. Therefore, Brpf1a is a developmental stage-specific chromatin regulator that complicates adult HSC homeostasis.

**Figure 7:**
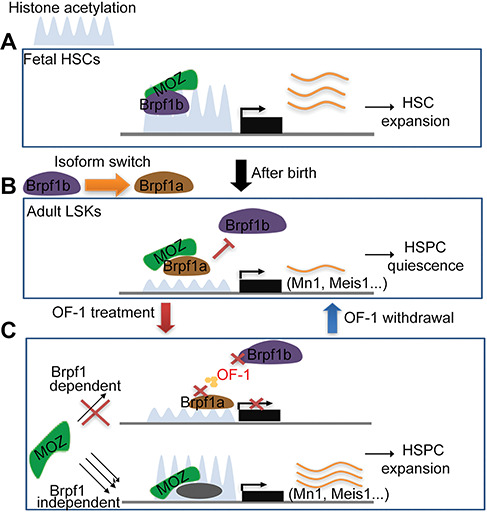
Proposed model of Brpf1 isoform switch during HSC development and OF-1-mediated adult HSC expansion enhancement. (**A**) In fetal liver, Brpf1b, which is mainly expressed in HSCs, combines with MOZ to modulate HSC development through promoting histone acetylation in downstream genes. (**B**) After birth, Brpf1a takes place of Brpf1b and becomes the most abundant Brpf1 isoform in HSCs in adult bone marrow. Brpf1a combines with MOZ to manipulate HSC quiescence through repressing histone acetylation in downstream genes *in vivo*. (**C**) The addition of OF-1 inhibits Brpf1a, which results in the release of MOZ. The free MOZ stimulates self-renewal-related genes to facilitate HSC self-renewal division. Moreover, OF-1 withdrawal *ex vivo* leads to the reactivation of Brpf1a and helps to regain the functional HSCs property.

A variety of small molecules have been explored to promote *ex vivo* expansion of HSPCs; however, acceleration of expansion is often associated with cellular stress and functional exhaustion of HSCs (Zhang and Gao, 2016; Ferreira and Mousavi, 2018). A potent strategy for HSPC expansion that activates both the Wnt/β-catenin pathway and the Akt or mTOR pathways may be associated with tumorigenesis (Zhang et al., 2006; Siegemund et al., 2015). SR1 (Gori et al., 2012) and UM171 (Fares et al., 2014) have been reported to expand human cord blood-derived short-term and long-term HSCs, respectively, but do so via an unknown mechanism. As there is no known clinical use for SR1 or UM171, there is a clinical need for new small molecules that enhance *ex vivo* expansion of HSPCs, particularly molecules that encourage *ex vivo* expansion without exerting a negative influence on the HSC multipotency. Histone acetylation determines transcriptional activity of many genes associated with HSC expansion and are also reversible, making them good candidates as therapeutic targets (De Felice et al., 2005; Nishino et al., 2011). Furthermore, acetyltransferases (e.g. MOZ) have been shown to regulate various stages of normal hematopoiesis. The current study showed that the OF-1 deserves attention as a candidate molecule for enhancing HSPC expansion because it increases self-renewing division without skewing differentiation division when the drug is withdrawn. Moreover, we clarified how OF-1 regulates self-renewing and differentiation division of LSKs at the molecular level. Transcriptome profiling revealed that OF-1 induced an MOZ-dependent gene activation program, which includes a set of master TFs known to be critical for HSC self-renewal (*Meis1* and *Mn1*) (Figure 7). OF-1 also facilitates an open chromatin state at self-renewal-related genes and sustains the open chromatin state of differentiated genes. OF-1 withdrawal caused activation of Brpf1a-mediated transcriptional program, reminiscent of differentiated LSKs (Figure 7). Thus, OF-1 acts as a reversible modulator that ensures functional expansion of adult HSC without exhaustion and multipotency loss.

HSCs undergo different developmental changes throughout life, although the transition from fetal to adult hematopoiesis is the most dramatic one. Fetal hematopoiesis, characterized by rapid proliferation of undifferentiated HSCs, supports embryo development. In adult stage hematopoiesis, most adult HSCs become quiescent, and blood cell production depends on the balance between HSC self-renewal and differentiation (Bernitz et al., 2016). Fetal HSCs and adult HSCs differ in expression of genes that are largely regulated by histone acetylation and chromatin organization. Previous reports showed that the Brd1/Brpf2 complex is responsible for global H3K14ac and is required for erythropoiesis in fetal HSCs (Mishima et al., 2011). The H4K16ac activity of the HAT MOZ is required for hematopoiesis in adult HSCs, but not early fetal and midgestational HSCs (Valerio et al., 2017). In our study, a genome-wide distribution of H3K27ac in adult hematopoietic cells at different stages illustrated that H3K27ac was a critical regulator of self-renewal-related gene transcription. We further showed that Brpf1a inhibition, but not Brpf1b, plays an important role in promoting adult LSK cell expansion. Based on the expression of Brpf1a and Brpf1b in fetal and adult HSCs, we conclude that there is a switch in the abundance of Brpf1 isoforms during HSC development. In adulthood, dominate Brpf1 isoform Brpf1a may replace Brpf1b or prevent Brpf1b from histone leading to hematopoiesis-related gene inactivation and HSC quiescence (Figure 7). The previous study has been revealed that GSK6853 and PFI-4 have high selectivity for the Brpf1b not Brpf1a (Bamborough et al., 2016; Meier et al., 2017). Therefore, GSK6853 and PFI-4 display adult HSPC expansion inhibition activity. Future studies will be required to test the different functions of Brpf1a and Brpf1b in fetal hematopoietic cells. Moreover, it would be interesting to explore the role of Brpf1b-mediated epigenetic activation in leukemia.

In summary, this study focused on the role of Brpf1 isoforms in HSPC expansion *ex vivo* and found that targeting Brpf1a by OF-1 enhances HSC expansion without affecting differentiation potential. Development of more effective and more highly specific Brpf1a inhibitors or combinatorial therapies with other agents may provide effective *ex vivo* HSPC expansion approaches in the future.

## Materials and methods

### Mice

C57BL/6 mice were purchased from Guangdong Medical Laboratory Animal Center. Male and female mice (6–8 weeks) were randomly used in the experiments. Ethics Committees of Zhongshan School of Medicine on Laboratory Animal Care approved all experimental protocols.

 To evaluate the effect of OF-1 on LSK cell proliferation *in vivo*, C57BL/6 mice were intraperitoneally (i.p.) injected with vehicle (DMSO) or OF-1 (25 mg/kg) every other day for 12 days. Relative and absolute numbers of LSKs from bone marrow and peripheral blood were analyzed after 2 weeks of the last i.p. injection by flow cytometry.

### Cell sorting and flow cytometry

Bone marrow cells were flushed from femur in DPBS, and red blood cells were lysed by 1× RBC lysis buffer. For cell sorting, the HSPC surface maker was defined as Lin^−^Sca-1^+^c-Kit^+^ by BD FACSAria III and Beckman Coulter MoFlo Astrios EQs. Flow cytometric analyses were performed by BD LSRFortessa. The surface maker for phenotyping analyses, anti-mouse lineage cocktail (anti-mouse CD3, clone 17A2; anti-mouse Ly-6G/Ly-6C, clone RB6-8C5; anti-mouse CD11b, clone M1/70; anti-mouse CD45R/B220, clone RA3-6B2; anti-mouse Ter-119/erythroid cells, clone TER-119), Ly6A/E (Sca-1, clone D7), CD117 (c-Kit, clone 2B8), CD34 (clone HM34), CD3 (clone 17A2), CD45R/B220 (clone RA3-6B2), CD41a (clone eBioMWReg30), CD48 (clone HM48-1), CD150 (clone TC15-12F12.2), CD11b (Mac-1) (clone M1/70), Ly6G/Ly6C (Gr-1, clone RB6-8C5), CD62L (clone MEL-14), CD4 (clone RM4-5/GK1.5), CD8α (clone 53-6.7), Ter-119 (erythroid, clone TER-119), F4/80 (clone BM8), CD127 (IL-7Rα, clone A7R34), Ly6C (clone HK1.4), Ly6G (clone 1A8), CD45.1 (clone A20), CD45.2 (clone 104), CD135 (clone A2F10), CD16/32 (clone 93), CD25 (clone 3C7), and CD44 (clone IM7) were purchased from BioLegend or eBioscience. For analyzing TF and histone acetylation level, LSKs were fixed and permeabilized with the intracellular staining buffer kit (eBioscience) followed by the manufacturer’s protocol. Cells were stained with histone acetylation (Cell Signaling Technology, 9927) and anti-MN1 antibody (Santa Cruz Biotechnology, 390869). EdU detection of cell proliferation were performed with EdU assay kit (RIBOBIO Company) followed by the manufacturer’s protocol. Briefly, after incubated with 50 μM EdU for 2 h, the proliferation of cells was analyzed by FACS cytometry. FACS data were analyzed with FlowJo software.

### Cell culture and screen of inhibitors

Freshly isolated sorted LSKs from mice were plated in StemSpan medium SFEM II (STEMCELL Technologies) with additional ingredients (50 ng/ml SCF, 50 ng/ml TPO, 50 ng/ml FL3 ligand, and 50 ng/ml IL-3) at 37°C in a humidified atmosphere of 5% CO_2_. LSKs were seeded at 2000–3000 cells per well in the presence of vehicle (DMSO) or inhibitors (all purchased from Selleck) with several concentrations in SFEM (STEMCELL Technologies) media. Relative and absolute numbers of LSKs were analyzed after 7 days of culture by flow cytometry. Human embryonic kidney 293T cells were cultured in DMEM medium with 10% FBS at 37°C in a humidified atmosphere of 5% CO_2_.

### Colony-forming cell assays

LSKs were plated in triplicate in methylcellulose-based medium with recombinant cytokines (without erythropoietin [EPO]) (STEMCELL Technologies, M3534) supplemented with EPO. Different types of CFUs were counted after culturing for 7–10 days.

### Small number of sorted cells for RNA-seq

Small number of sorted LSKs (10–20 cells) were treated with vehicle (DMSO) or OF-1 (10 μM) for 7 days and then subjected for RNA-seq by ANOROAD GENOME.

### ATAC-seq chromatin preparation

LSKs (10000–20000 cells) were treated with vehicle (DMSO) or OF-1 (10 μM) for 7 days and then sorted by FACS. Library amplification was performed with the NEBnext High Fidelity 2× PCR Master Mix (M0541S, New England Biolabs). Prepared ATAC-seq libraries were sequenced with single-end 50-bp reads on the HiSeq 2000 platform (Suganuma et al., 2016). Raw reads were adaptor-trimmed and aligned to the genome. Peaks were called using the MACS2 software (v2.1.0.20140616) with default parameters.

### ChIP-seq

LSKs (1 × 10^6^) were treated with vehicle (DMSO) or OF-1 (10 μM) for 7 days and then sorted and cross-linked with 1% formaldehyde for 5 min at room temperature. ChIP-seq of these samples were prepared using a protocol as previously described. ChIP-qualified anti-Histone H3 (acetyl K9, K14, K18, K23, and K27)) antibody (Abcam, 47915) or rabbit IgG (Cell Signaling Technology) were used in ChIP-seq assays.

### Plasmid construction of shRNA-mediated knockdown and overexpression

The full-length sequence of mouse Brpf1 isoform 4 (Brpf1a) was generated and cloned to a lentiviral expression vector pTSB02, and isoform 1 (Brpf1b) was generated by Bridge PCR and then cloned to same lentiviral expression vector.

Designing and cloning of shRNA expression cassette into pLKO lentiviral vector followed the protocol. The shRNA sequences against Brpf1a and Mn1 were designed and cloned into the pLKO lentiviral vector. To generate lentiviral particles, the constructed shRNA expression plasmid was co-transfected with packaging plasmids psPAX2 and pVSVG into human embryonic kidney 293T cells using StarFect High-efficiency Transfection Reagent (Genstar). All used plasmids were confirmed by sequencing.

### qPCR analysis

Total RNA was extracted with RNeasy (Qiagen) and used to generate first-strand cDNA with the Superscript III cDNA synthesis system (Invitrogen) following the manufacturer’s instructions. qPCR analysis was performed using SYBR PrimeScript Ready Mix (TaKaRa) in an ABI 7900 sequence detection system (Applied Biosystems). GAPDH expression was used for normalization. The PCR primers are listed in Supplementary Table S2.

### Infection of mouse LSKs with lentivirus

Before lentiviral infection, sorted LSKs were planted in 6-well plate with a cytokine cocktail for several hours. For infection, cells were incubated with the lentivirus contained with Polybrene (10 μg/ml) and centrifuged at 1000 *g* for 90 min at room temperature and then cultured at 37°C for 6–8 h, then we removed unbound virus by changing medium. Positive cell selection was performed with 2 μg/ml puromycin for the 2–5 days post-infection. Infection efficiency of lentivirus-transduced LSKs were analyzed by flow cytometry or qPCR.

### LTC-IC assay

Limiting dilution of LTC-ICs was performed in 96-well format in Myelocult M5300 medium (STEMCELL Technologies, M5300). Weekly half medium changes were made for 5 weeks after which methylcellulose (STEMCELL Technologies, M3434) were added. LTC-IC frequency was calculated using ELDA (http://bioinf.wehi.edu.au/software/elda/).

### Extreme limiting dilution reconstitution assay

Competitive reconstitution assays were performed by intravenous transplantation of 1 × 10^5^, 5 × 10^4^, or 2 × 10^4^ donor-derived cells from DMSO- or OF-1-treated LSKs (CD45.2), together with 2 × 10^4^ rescue cells (CD45.1) into groups of six lethally X-ray-irradiated (9 Gy) recipient mice (CD45.1). The rescue cells (CD45.1) are bone marrow cells that can support the survival of transplanted cells (CD45.2). HSC frequencies were measured using ELDA (http://bioinf.wehi.edu.au/software/elda/) in which successful engraftment was defined as the presence of a distinct CD45.2^+^CD45.1^−^ population ≥ 5% of total hematopoietic cells in bone marrow.

### Statistical analysis

Statistical analysis was performed with Student’s *t*-test or analysis of variance, two-tailed using SPSS 11.5. Sample sizes are indicated in figure legends. All data are presented as mean ± SD, and the significant differences are shown as *P < 0.05, **P < 0.01, and ***P < 0.001.

## Supplementary Material

Supplemental_information_F_mjz092
