# Supplementary material for: Isoform-specific involvement of Brpf1 in expansion of adult hematopoietic stem and progenitor cells
Source: J Mol Cell Biol. 2019 Sep 30;12(5):359–71. doi: 10.1093/jmcb/mjz092 (PMC7288741; doi:10.1093/jmcb/mjz092)
Supplement: Supplemental_information_F_mjz092 [file supplemental_information_f_mjz092.pdf]

Supplementary Information

Isoform-specific involvement of Brpf1 in expansion of adult hematopoietic stem and progenitor cells

Qiuping He, Mengzhi Hong, Jincan He, Weixin Chen, Meng Zhao, and Wei Zhao

Contents

Supplemental Figures 1-5

Supplemental Table 1-3

Supplemental Figures

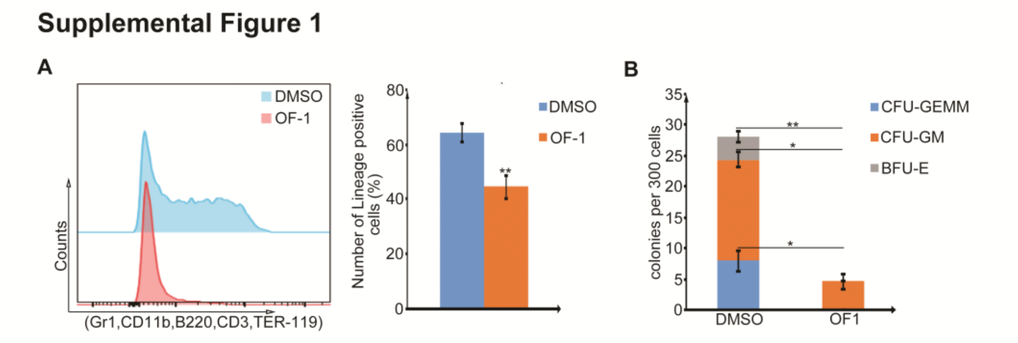

Supplemental Figure 1. OF-1 impairs LSKs differentiation and colony formation. Related to Figure 2.

(A) Percentage of Lineage (Gr1,CD11b, B220, CD3 and TER-119) positive cells upon 10 μM OF-1treatment. Representative FACS profiles are shown on the left, and the percentage of positive cells is shown on the right.

(B) In vitro colony assay after 10 μM OF-1 treatment. Erythroid progenitors (BFU-E), granulocyte-macrophage progenitors (CFU-GM), and multi-potential granulocyte, erythroid, macrophage, and megakaryocyte progenitors (CFU-GEMM) colony-forming ability was analyzed.

Data represent mean ± SD from three independent experiments, with statistical analysis defined by two-tailed Student's t test: $*p < 0.05$; $**p < 0.01$; n.s., not significant.

Supplemental Figure 2

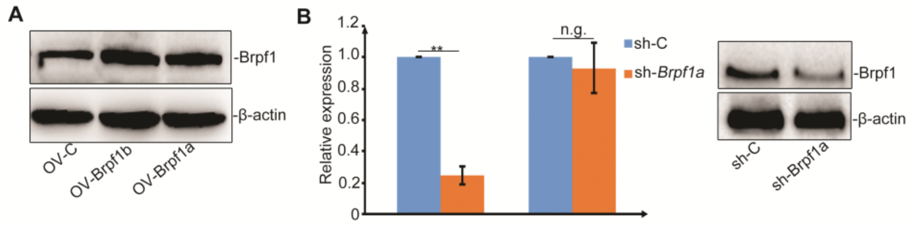

Supplemental Figure 2. Brpf1a or Brpf1b overexpression and Brpf1a knockdown. Related to Figure 3.

(A) Immunoblotting analysis of Brpf1 protein in LSK cells transfected with Brpf1a-FLAG or Brpf1b-FLAG.

(B) qPCR analysis of Brpf1a and Brpf1b mRNA levels in Brpf1a KD LSKs (Left). Immunoblotting analysis of Brpf1 protein in LSK cells transfected with sh-C or sh-Brpf1a (Right).

Data represent mean ± SD from three independent experiments, with statistical analysis defined by two-tailed Student's t test: $*p < 0.05$; $**p < 0.01$; n.s., not significant.

Supplemental Figure 3

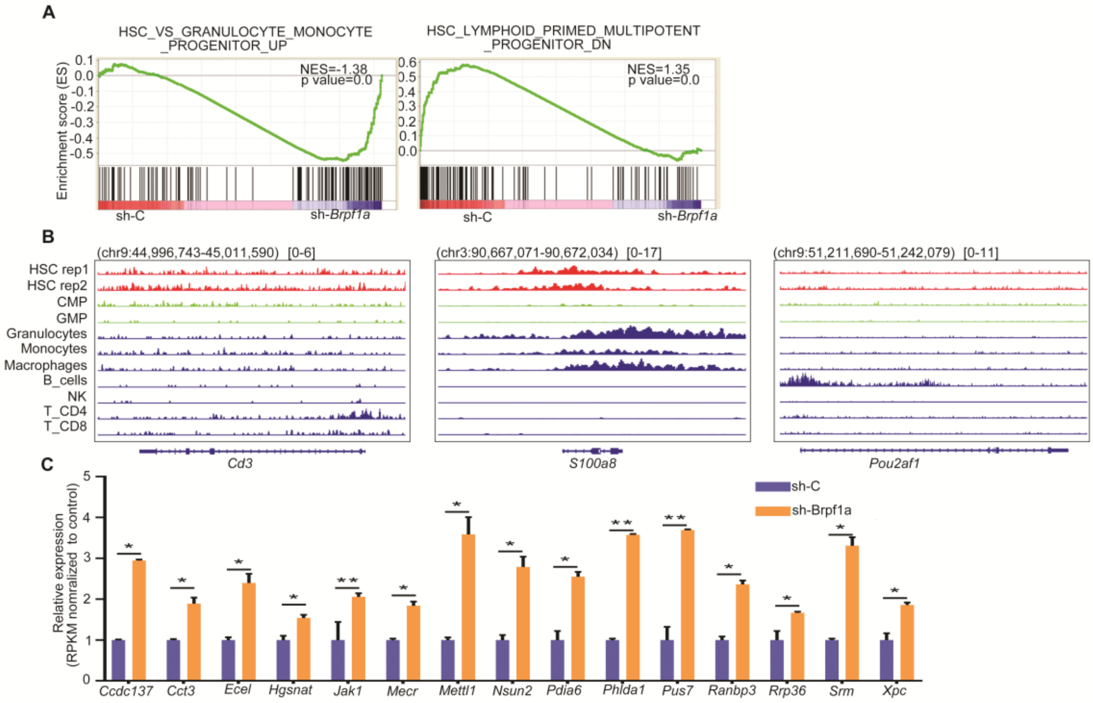

Supplemental Figure 3. Repression of Brpf1a leads to increased expression of multiple genes critical for HSC self-renewal. Related to Figure 4.

(A) GSEA reveals enrichment of the differentiation signature in Brpf1a KD regulated genes of LSKs.

(B) Integrative Genomics Viewer (IGV) view showing the ChIP-seq of H3K27ac profiles (cell types annotated at left) in representative “differentiation” genes. Data are from GEO dataset (GSE60101).

(C) Relative mRNA expression levels of represented genes related to HSC self-renewal in Brpf1a KD and control LSKs.

Data represent mean ± SD from three independent experiments, with statistical analysis defined by two-tailed Student's t test: ∗$p < 0.05$; ∗∗$p < 0.01$; n.s., not significant.

Supplemental Figure 4

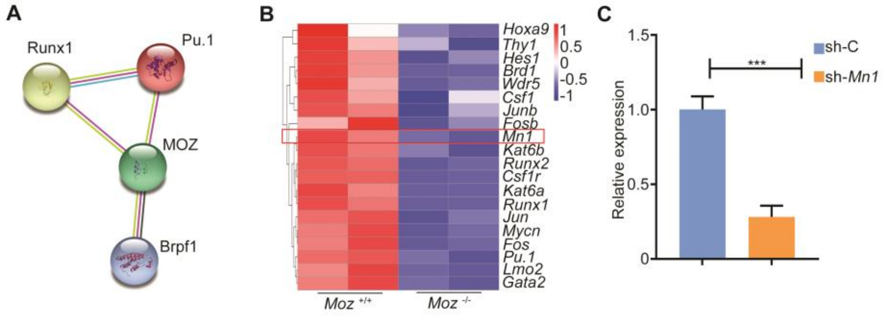

Supplemental Figure 4. MOZ cooperates with Brpf1 playing a crucial role in hematopoiesis. Related to Figure 5.

(A) Using the STRING online database, some self-renewal-related TFs were filtered into the PPI network.

(B) Heatmap of hematopoietic genes expression levels in the LSKs from *Moz*$^{+/+}$ and *Moz*$^{-/-}$ mice.

(C) qPCR analysis of Mn1 mRNA levels in Mn1 KD LSKs.

Data represent mean ± SD from three independent experiments, with statistical analysis defined by two-tailed Student's t test: ∗p < 0.05; ∗∗p < 0.01; n.s., not significant.

Supplemental Figure 5

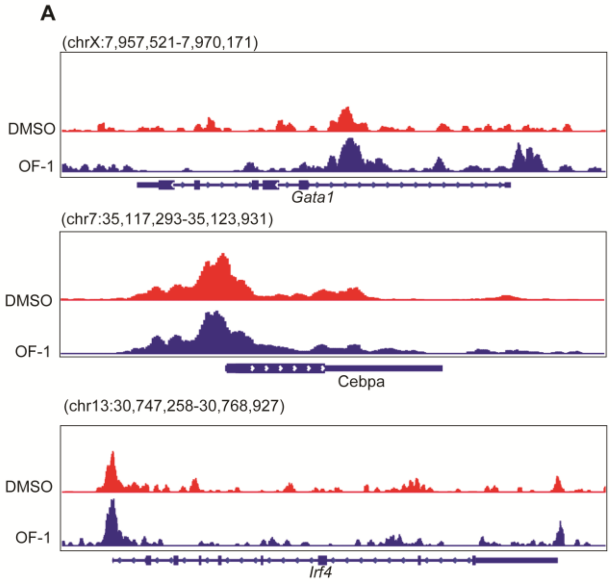

Supplemental Figure 5. DNA accessibility unchanged of lineage differentiation genes. Related to Figure 6.

(A) Genome browser tracks of DMSO and OF-1 treated LSK cells ATAC-seq data in Gata1, Cebpa and Irf4 loci.

Supplemental Table 1. The inhibitor information.

inhibitor	formula	chemical structure	target
OF-1	C17H18BrN3O4S	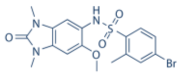	BRPF1B,BRPF2
GSK6853	C22H27N5O3	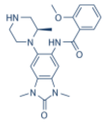	BRPF1
PFI-1	C16H17N3O4S	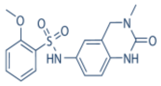	BRD4,BRD2
RVX-208	C20H22N2O5	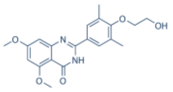	BRD2
I-BET151	C23H21N5O3	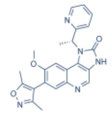	BRD2
GSK1324726A	C25H23ClN2O3	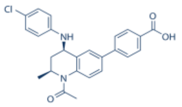	BRD2,BRD3,BRD4
(+)-JQ1	C23H25ClN4O2S	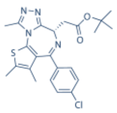	BRD4(1/2)
CPI-0610	$C_{20}H_{16}ClN_3O_2$	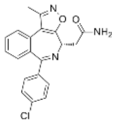	BRD4-BRD1
PFI-4	C21H24N4O3	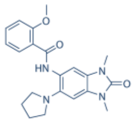	BRPF1

Supplemental Table 2. The PCR primers used in quantitative PCR assay.

**gene**	**Primer name**	**qPCR primer sequence (5’→3’)**
Brpf1a	*Brpf1a* F	ACCGAATTGGACGAAGTACC
	Brpf1a R	GCACTGCTGCCCTGTAGAAG
Brpf1b	*Brpf1b* F	CCAGCACAACTCCCTCAA
	Brpf1b R	CAGCACCCCTGACTTTGG
Gapdh	*Gapdh* F	TCCCACTCTTCCACCTTCGATGC
	Gapdh R	GGGTCTGGGATGGAAATTGTGAGG
Hoxb5	*Hoxb5* F	CCTTCTCGGGGCGTTATCC
	Hoxb5 R	CCTGAAGCGGGGTTCCTTG
Hoxa9	*Hoxa9* F	CCCCGACTTCAGTCCTTGC
	Hoxa9 R	GATGCACGTAGGGGTGGTG
Mn1	*Mn1* F	AGATCCAGCTGCAGAGACAA
	Mn1 R	TACTCATGGCGCTCTTGACT

Supplemental Table 3. The shRNA sequences.

**gene**	**Primer name**	**shRNA primer sequence (5'→3')**
Brpf1a	*Brpf1a* F	CCGGGTAACCGAATTGGACGAAGTACTCGAGTA CTTCGTCCAATTCGGTTACTTTTTG
	Brpf1a R	AATTCAAAAAGTAACCGAATTGGACGAAGTACT CGAGTACTTCGTCCAATTCGGTTAC
Mn1	*Mn1* F	CCGGCCGGGACCTTCTTCGACAAATCTCGAGAT TTGTCGAAGAAGGTCCCGGTTTTTG
	Mn1 R	AATTCAAAAACCGGGACCTTCTTCGACAAATCT CGAGATTTGTCGAAGAAGGTCCCGG
